# Targeting Dopamine Receptor D2 by Imipridone Suppresses Uterine Serous Cancer Malignant Phenotype

**DOI:** 10.3390/cancers12092436

**Published:** 2020-08-27

**Authors:** Wen Hu, Li Zhang, Sammy Ferri-Borgogno, Suet-Ying Kwan, Kelsey E. Lewis, Han T. Cun, Tsz-Lun Yeung, Pamela T. Soliman, Rohinton S. Tarapore, Joshua E. Allen, Xinyuan Guan, Karen H. Lu, Samuel C. Mok, Chi-Lam Au-Yeung

**Affiliations:** 1 Department of Gynecologic Oncology and Reproductive Medicine, The University of Texas MD Anderson Cancer Center, Houston, TX 77030, USA; whu2@mdanderson.org (W.H.); lzhang25@mdanderson.org (L.Z.); sferri@mdanderson.org (S.F.-B.); hcun@mdanderson.org (H.T.C.); tyeung@mdanderson.org (T.-L.Y.); psoliman@mdanderson.org (P.T.S.); khlu@mdanderson.org (K.H.L.); scmok@mdanderson.org (S.C.M.); 2 State Key Laboratory of Oncology in South China and Collaborative Center for Cancer Medicine, Sun Yat-Sen University Cancer Center, Guangzhou 510060, China; xyguan@hkucc.hku.hk; 3 Department of Molecular and Cellular Biology, The University of Texas MD Anderson Cancer Center, Houston, TX 77030, USA; skwan1@mdanderson.org; 4 Department of Obstetrics and Gynecology, The University of Texas Medical Branch at Galveston, Galveston, TX 77555, USA; kelewis@utmb.edu; 5 Oncoceutics Inc., Philadelphia, PA 19104, USA; rohinton.tarapore@oncoceutics.com (R.S.T.); josh.allen@oncoceutics.com (J.E.A.)

**Keywords:** uterine serous cancer, imipridone, dopamine receptor D2, metabolic reprogramming

## Abstract

Uterine serous cancer (USC) is an aggressive subtype of endometrial cancer, with poor survival and high recurrence rates. The development of novel and effective therapies specific to USC would aid in its management. However, few studies have focused solely on this rare subtype. The current study demonstrated that the orally bioavailable, investigational new drug and novel imipridone ONC206 suppressed USC cell proliferation and induced apoptosis both in vitro and in vivo. Disruption of the DRD2-mediated p38MAPK/ERK/PGC-1α network by ONC206 led to metabolic reprogramming and suppression of both glycolysis and oxidative phosphorylation. ONC206 also synergized with paclitaxel in reducing USC cell viability. In addition, DRD2 overexpression correlated with poor overall survival in patients. This study provides the first evidence that ONC206 induced metabolic reprogramming in USC cells and is a promising therapeutic agent for USC treatment. These findings support further development of ONC206 as a promising therapeutic agent and improves survival rates in patients with USC.

## 1. Introduction

Uterine serous cancer (USC) is an aggressive subtype of endometrial cancer, which is one of the most common cancers in women in the United States [1]. Although USC accounts for less than 10% of endometrial malignancies, it causes a disproportionate 40% of deaths related to endometrial cancer [2]. Currently, the most common management strategy for USC is hysterectomy and surgical staging, followed by platinum/paclitaxel-based chemotherapy and radiation therapy [3,4,5,6,7]. However, most USC patients have an unfavorable 5-year survival rate (41%) and poor outcomes due to high recurrence rates and limited effective treatment for recurrence [8,9,10,11]. Thus, new therapeutic strategies to manage USC are needed.

Small molecules have been developed to induce endogenous genes, such as tumor necrosis factor-related apoptosis-inducing ligand (TRAIL), to serve as promising therapeutic approaches. A novel orally bioavailable small molecule, ONC201 (initially called TRAIL-inducing compound 10, or TIC10), was identified as the first-in-class imipridone that was shown to have anti-tumor effects through continuous dual induction of the endogenous TRAIL tumor suppressor and its pro-apoptotic receptor DR5 [12]. In addition to its TRAIL-inducing property, ONC201 has been shown to selectively antagonize dopamine receptors of the D2-like class including DRD2 and DRD3, which mediates the effect of dopamine and is coupled to Gi subtype of G-protein-coupled receptor (GPCR) using computational methods [13,14]. In vitro experiments showed that ONC201 is a direct competitive antagonist of DRD2 and DRD3, with a Ki of 3 μM [15]. GPCRs are reported to control critical pro-survival and stress-signaling pathways that are often dysregulated in human cancer to favor cancer cell survival and progression [16], and DRD2 are often overexpressed by tumor cells and can be activated by dopamine produced in its tumor microenvironment or by the tumor cells themselves [14].

To improve the clinical efficacy of ONC201 and extend the use of imipridones in the clinic, researchers have developed a series of chemical derivatives of ONC201 based on the unique imipridone core, representing new compounds with better therapeutic efficacies to target ONC201-resistant tumors [17]. Among the analogs, ONC206, a difluorobenzyl imipridone, demonstrated a distinct efficacy spectrum and favorable therapeutic potency, thus qualifying for further study [17]. In previous studies, ONC206 was reported to exhibit anti-tumor effects in a colon cancer model and a glioblastoma model via dual inhibition of the AKT and ERK signaling pathways [17,18]. Moreover, GPCR profiling with β-arrestin recruitment revealed that although ONC206 selectively antagonized DRD2/3 like ONC201, it exhibited a much lower Ki value (~320 nM) than ONC201 for DRD2 with specificity across human GPCRs and complete antagonism [19]. Schild analyses of ONC206 in cAMP and β-arrestin recruitment assays also revealed hallmarks of noncompetitive DRD2 antagonism, unlike antipsychotics but similar to ONC201 [19]. Besides, shotgun mutagenesis across DRD2 identified seven residues critical for ONC206-mediated antagonism at orthosteric and allosteric sites. The impact and magnitude of different mutants varied between ONC201 and ONC206 and one of the allosteric residues was unique to ONC206 [19]. These suggested that the tumor suppressing effect of ONC206 might be stronger than ONC201.

Additionally, the US Food and Drug Administration has recently accepted ONC206 as an investigational new drug, allowing the first-in-human phase I trial of the compound in adults with primary central nervous system neoplasms, at the National Cancer Institute. Despite these studies, the therapeutic efficacy of ONC206 in USC, as well as the molecular mechanism by which ONC206 suppresses USC progression, has not been explored.

In the current study, we sought to determine the therapeutic properties of ONC206 in USC, as well as identify the distinct signaling networks associated with the anti-tumor effects of ONC206. A greater understanding of the anti-tumor effects of ONC206 in USC and the pathways involved may lead to novel therapeutic strategies for the treatment of USC.

## 2. Results

### 2.1. ONC206 Inhibits Cell Proliferation and Induces Apoptosis

**Figure 1:**
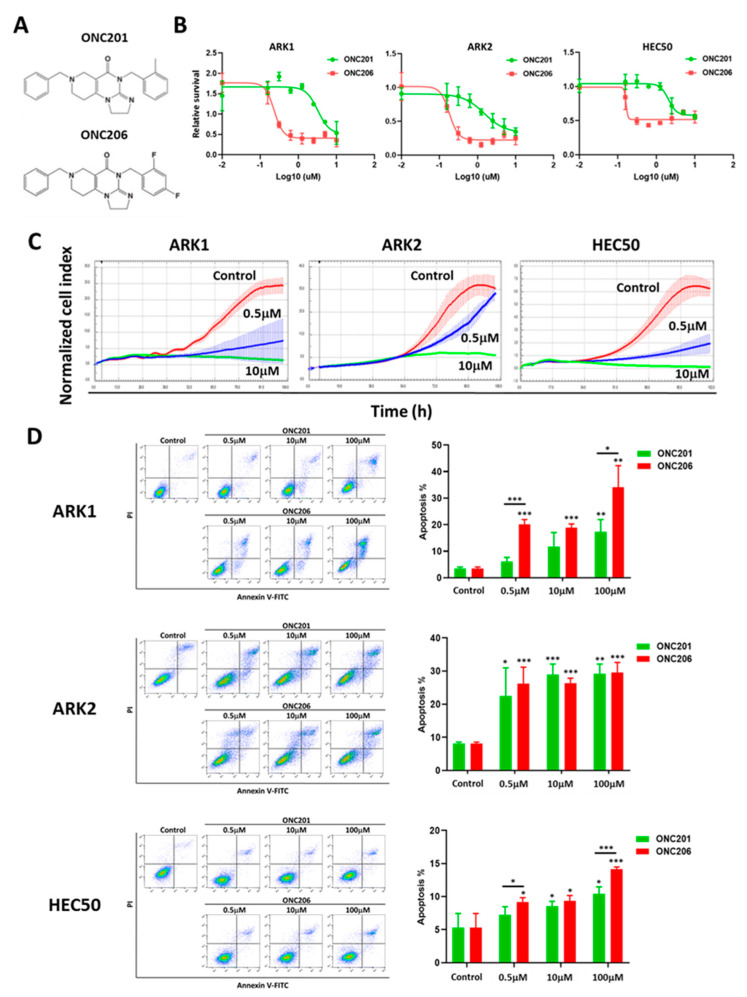
Effect of ONC206 on uterine serous cancer cell viability and apoptosis in vitro. (**A**) Chemical structure of imipridones ONC201 and ONC206. (**B**) ONC201 and ONC206 suppress cell viability of ARK1, ARK2, and HEC50 cells after 72 h of treatment, as measured using the MTT assay. Three independent experiments were performed (mean ± standard deviation). (**C**) ONC201 and ONC206 (0.5 and 10 μM) suppress cell proliferation of ARK1, ARK2, and HEC50 cells, as measured using the xCELLigence real-time cell analysis assay. (**D**) ONC201 and ONC206 (0.5, 10 and 100 μM) induce cell apoptosis of ARK1, ARK2, and HEC50 cells after 72 h of treatment, as measured using Annexin V-fluorescein isothiocyanate (FITC) and propidium iodide (PI) staining. Representative images are shown. Results in the bar charts were averaged from three independent experiments and are shown as mean ± standard deviation. *** *p* < 0.001, ** *p* < 0.01, * *p* < 0.05, two-tailed Student t test.

To determine the tumor suppressive effect of ONC206 on human USC cells and compare it with that of its parent compound ONC201 (Figure 1A), we treated USC cells ARK1, ARK2 and HEC50 with different concentrations of ONC201 and ONC206 for 72 h before cell viability was measured using the MTT assay. The results showed that both ONC201 and ONC206 suppressed USC cell viability in a dose-dependent manner and the effect of ONC206 was markedly stronger than that of ONC201 (Figure 1B). IC50 values of ONC206 in ARK1, ARK2 and HEC50 cells were 4-to 10-fold lower than those of ONC201 (Table S1).

Next, the xCELLigence real-time cell analysis system was used to monitor USC cell growth in response to treatment with ONC206. The results showed that ONC206 suppressed cell proliferation of ARK1, ARK2 and HEC50 cells in a dose-dependent manner (Figure 1C). We also determined whether ONC206 induced apoptosis in USC cells. ARK1, ARK2 and HEC50 cells were exposed to 0.5, 10 and 100 μM ONC201 or ONC206 for 72 h and the number of apoptotic cells was detected using Annexin V and propidium iodide staining. The results showed that both ONC201 and ONC206 induced apoptosis in USC cells (Figure 1D).

### 2.2. ONC206 Reduces Uterine Tumor Burden in Xenograft Mouse Model

**Figure 2:**
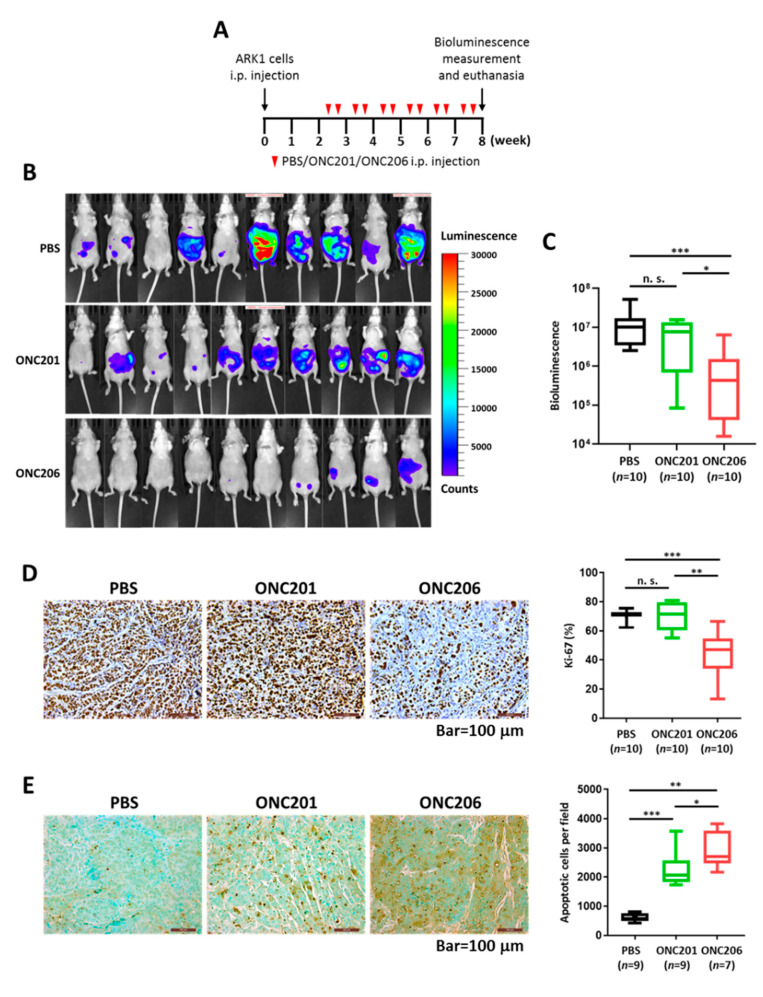
Effect of ONC206 on uterine tumor growth and apoptosis in vivo. (**A**) Schema shows the use of an in vivo model to evaluate the effect of ONC206 on uterine tumor growth. i.p., intraperitoneal; PBS, phosphate-buffered saline. (**B**) Images show lower bioluminescence signals in ARK1 cell-bearing nude mice treated with ONC201 (*n* = 10) or ONC206 (*n* = 10) than in untreated PBS controls (*n* = 10). (**C**) Box plot shows a significantly lower bioluminescence from ARK1 cell-bearing nude mice treated with ONC206 (*n* = 10) than those treated with ONC201 (*n* = 10; *p* < 0.05, Mann–Whitney U test) or untreated PBS controls (*n* = 10; *p* < 0.001, Mann–Whitney U test). (**D**,**E**) Representative microscopic images of paraffinized sections of tumor tissues collected from ARK1 cell-bearing nude mice 6 weeks after treatment with ONC201 or ONC206, showing significantly (**D**) lower Ki-67 expression and (**E**) higher apoptosis in the ONC206 treatment group compared with the ONC201 treatment group or untreated controls. Bar = 100 μm. Quantification of cell staining positively for Ki-67 or apoptotic cells for each group is shown in the box plot. *** *p* < 0.001, ** *p* < 0.01, * *p* < 0.05, n. s. = not significant (*p* > 0.05), Mann-Whitney U test.

To determine the tumor-suppressive effect of ONC206 in vivo, we established a xenograft mouse model by injecting luciferase-labeled ARK1 cells into female athymic mice intraperitoneally. The mice were then injected with 100 mg/kg ONC201 or ONC206 intraperitoneally twice per week for 6 weeks (Figure 2A). Tumor growth was monitored by the IVIS-Lumina XR in vivo imaging system. The results showed that mice treated with ONC206 had significantly lower bioluminescent signals than those treated with PBS or ONC201 (Figure 2B,C). In addition, we did not observe any body weight loss among any of the groups, suggesting that the therapeutic dose of drugs used were tolerable in athymic mice (Figure S1).

Next, we determined the number of proliferating and apoptotic cells in tumor tissues from mice using immunohistochemistry. Proliferating tumor cells were quantified by the number of cells staining positive for Ki-67. The results showed that tumor tissues from ONC206-treated mice had significantly fewer Ki-67-positive cells than those from ONC201-treated or control untreated mice (Figure 2D). Apoptotic cells were quantified using the TUNEL method. The results showed that tumor tissues from ONC206-treated mice had significantly more apoptotic cells than those from ONC201-treated or control untreated mice (Figure 2E). These findings suggested that ONC206 has higher efficacy in suppressing USC progression than ONC201 in vivo.

### 2.3. ONC206 Suppresses USC Progression through the p38MAPK/ERK Pathways and Metabolic Reprogramming

**Figure 3:**
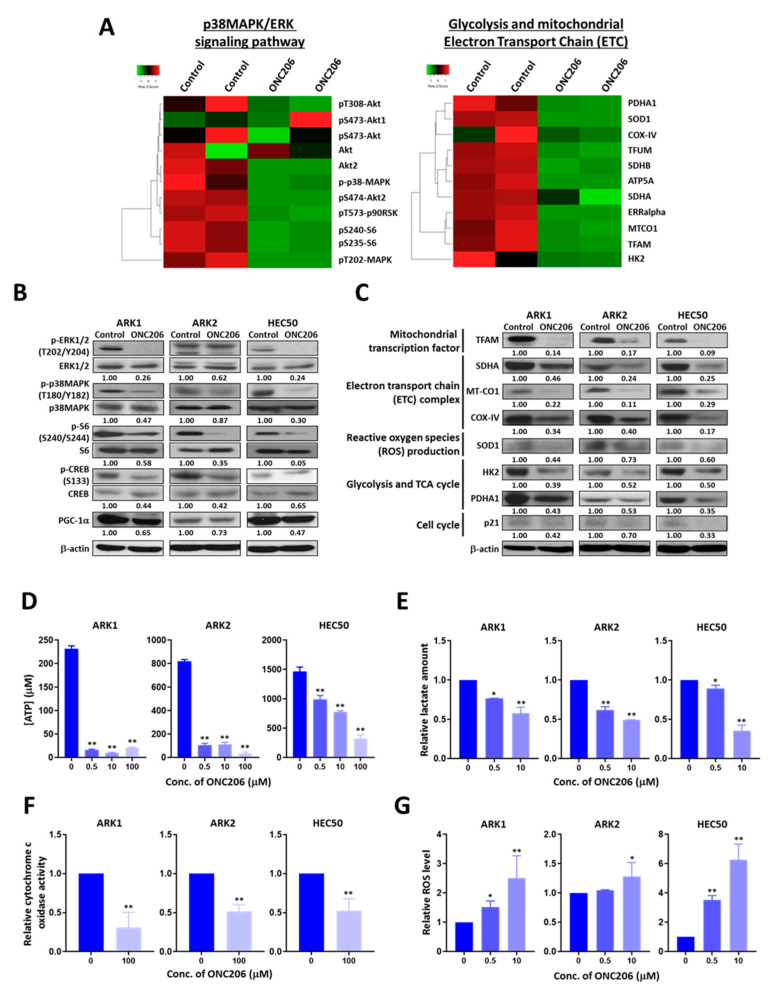
Effect of ONC206 on mitochondrial functions and glycolysis in uterine serous cancer cells. (**A**) Heat maps obtained using reverse phase protein array analysis shows differentially expressed proteins related to the p38MAPK/ERK signaling network and mitochondrial functions in ARK1 cells with (*n* = 2) or without (*n* = 2) 48 h of treatment with 50 μM ONC206. (**B**) Western blot analyses show decreased protein levels of p-ERK1/2, p-p38MAPK, p-S6, p-CREB, and PGC-1α in ONC206-treated ARK1, ARK2 and HEC50 cells compared with control cells without treatment. β-actin served as a loading control. Ratios between phosphorylated and total protein levels are presented. Three independent experiments were performed. (**C**) Western blot analyses show lower protein levels of TFAM, SDHA, MT-CO1, COX-IV, SOD1, HK2, PDHA1, and p21 in ONC206-treated ARK1, ARK2, and HEC50 cells compared with control cells without treatment. β-actin served as a loading control. Relative normalized protein levels with respect to the corresponding control are presented. Three independent experiments were performed. (**D**) ATP production as measured by the ATP production kit, (**E**) lactate secretion as measured by the Lactate-Glo assay kit, and (**F**) cytochrome c oxidase activity as measured by the cytochrome c oxidase assay kit were decreased in ARK1, ARK2, and HEC50 cells after treatment with ONC206. Results were averaged from three independent experiments and are shown as mean ± standard deviation. ** *p* < 0.01, * *p* < 0.05, two-tailed Student *t* test. (**G**) Reactive oxygen species (ROS) productions was increased in ARK1, ARK2, and HEC50 cells after 24 h of treatment with ONC206 as measured using the ROS detection assay kit. Results were averaged from three independent experiments and are shown as mean ± standard deviation. ** *p* < 0.01, * *p* < 0.05, two-tailed Student *t* test.

To determine the molecular mechanisms by which ONC206 suppressed USC, we performed RPPA analyses for more than 400 proteins associated with key signaling pathways in ONC206-treated ARK1 cells. Differential expression of proteins in the p38MAPK/ERK signaling pathways, the mitochondrial electron transport chain (ETC) and apoptosis were observed (Figure 3A and Figure S2A, Table S2).

The RPPA results were then validated using Western blot analyses on USC cells ARK1, ARK2 and HEC50 treated with ONC206 or the control buffer. The results showed that ONC206-treated cells had markedly lower levels of phospho-p38MAPK, phospho-ERK, phospho-S6 and peroxisome proliferator activator receptor gamma coactivator 1 alpha (PGC-1α), suggesting that the p38MAPK/ERK/PGC-1α signaling pathways are inactivated in ONC206-treated cells (Figure 3B). We also observed significantly lower expression of key proteins associated with glycolysis, such as hexokinase II (HK2) and pyruvate dehydrogenase E1 subunit alpha 1 (PDHA1), as well as those associated with the mitochondrial ETC, including mitochondrially encoded cytochrome c oxidase 1 (MT-CO1), succinate dehydrogenase complex flavoprotein subunit A (SDHA), cytochrome c oxidase subunit 4 (COX-IV) and mitochondrial transcription factor A (TFAM), and those associated with ROS production, such as superoxide dismutase 1 (SOD1), in ONC206-treated USC cells compared with control cells, suggesting that ONC206 plays a role in metabolic reprogramming (Figure 3C).

In contrast, expression levels of pro-apoptotic cleaved caspase 3 were markedly higher in ONC206-treated cells than in control cells, suggesting that ONC206 plays a role in apoptosis, as we described above (Figure S2B). We did not observe a significant induction of TRAIL expression in USC cells after treatment with ONC206, as previously reported in ONC201-treated USC cells (Figure S2C) [12].

### 2.4. ONC206 Suppresses Glycolysis and Oxidative Phosphorylation (OXPHOS) in USC Cells

The RPPA and Western blot results indicated that ONC206 might induce metabolic reprogramming by altering the expression of proteins associated with glycolysis and the mitochondrial ETC. To further evaluate the effects of ONC206 on metabolic reprogramming, we determined ATP production, cytochrome c oxidase activity, ROS production and lactate production in USC cells after treatment with ONC206. The results showed that ARK1, ARK2 and HEC50 cells treated with ONC206 had significantly lower levels of ATP and lactate production compared with control cells, suggesting that ONC206 suppresses both glycolysis and OXPHOS in USC cells (Figure 3D,E). We also observed a decrease in cytochrome c oxidase activity and an increase in ROS production in ONC206-treated ARK1, ARK2 and HEC50 cells compared with control cells, suggesting a potential mechanism by which ONC206 induces apoptosis in USC cells (Figure 3F,G).

### 2.5. DRD2 Mediates the Tumor-Suppressive Effect of ONC206 in USC Cells

**Figure 4:**
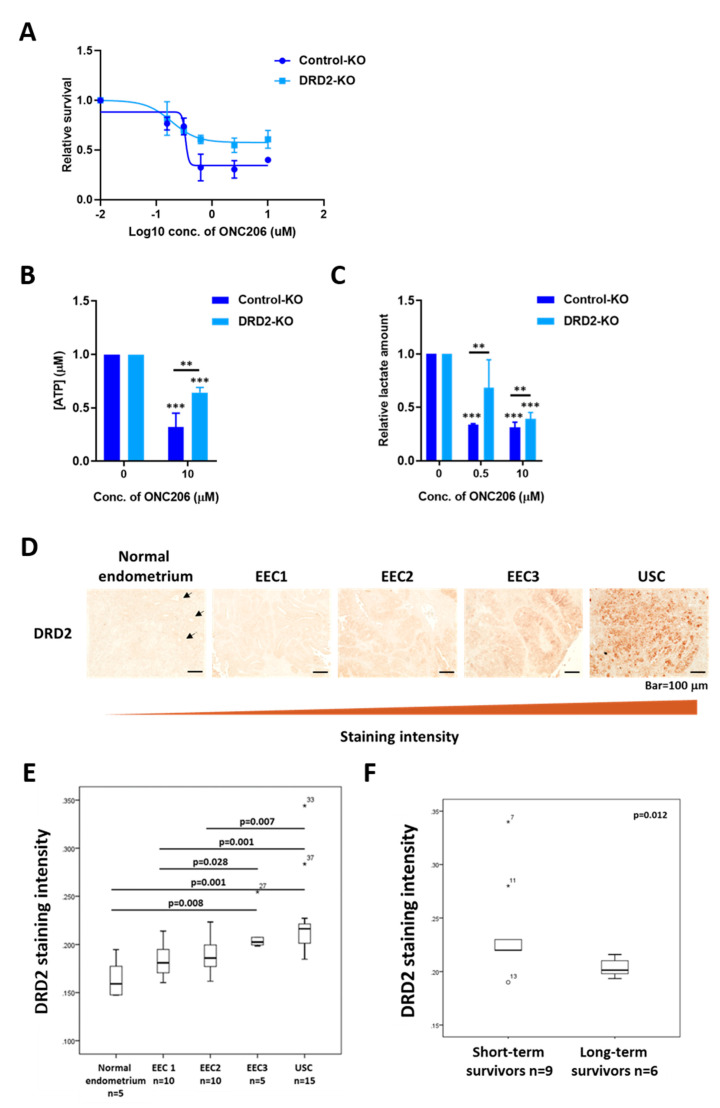
Role of DRD2 in mediating the effect of ONC206 on uterine serous cancer (USC) cells. (**A**) DRD2 knockout (DRD2-KO) ARK2 cells are more resistant to ONC206 than control knockout (Control-KO) ARK2 cells, as measured by the MTT assay. Three independent experiments were performed (mean ± standard deviation). (**B**) Decrease in ATP production in DRD2-KO ARK2 cells after 48 h of treatment with ONC206 as measured by the ATP determination kit. Results were averaged from three independent experiments and are shown as mean ± standard deviation. *** *p* < 0.001, ** *p* < 0.01, two-tailed Student t test. (**C**) Decrease in lactate secretion in DRD2-KO ARK2 cells after 24 h of treatment with ONC206 as measured by the Lactate-Glo kit. Results were averaged from three independent experiments and are shown as mean ± standard deviation. *** *p* < 0.001, ** *p* < 0.01, two-tailed Student t test. (**D**) Representative microscopic images of paraffinized sections of different stages of gynecological tissues including normal endometrium, endometrioid endometrial cancer (EEC; grades 1 to 3) and uterine serous cancer (USC). An increase in DRD2 expression from normal endometrium to USC is shown. Bar = 100 μm. Arrows indicate the uterine gland structures in normal endometrium. (**E**) Quantification of DRD2 staining intensity for normal endometrium (*n* = 5), grade 1 EEC (*n* = 10), grade 2 EEC (*n* = 10), grade 3 EEC (*n* = 5) and USC (*n* = 15) is shown in the box plot. (**F**) Box plot shows a significant decrease in DRD2 expression in long-term USC survivors (>5 years; *n* = 6) compared with short-term survivors (<2 years; *n* = 9; *p* = 0.012, Mann–Whitney U test).

Because ONC201 has been shown to bind to and selectively antagonize DRD2 [20], we hypothesized that the tumor-suppressive effects of ONC206 are also mediated by DRD2. We first determined the effect of DRD2 silencing on the tumor-suppressor effect of ONC206. DRD2 was successfully knocked out in ARK2 cells using CRISPR/Cas9 technology (Figure S3A,B). Control-KO and DRD2-KO cells were treated with various concentrations of ONC206. The results showed that the effect of ONC206 on cell viability was smaller in DRD2-KO ARK2 cells compared with Control-KO cells. Meanwhile, Control-KO cells showed a similar response to that of the parental ARK2 cells, suggesting that DRD2 mediates the tumor-suppressor effect of ONC206 and USC cells expressing higher levels of DRD2 are more sensitive to ONC206 (Figure 4A and Figure S3C). DRD2-KO cells had lower cell viability than Control-KO cells even without treatment with ONC206, suggesting that DRD2 plays an important role in USC progression (Figure S3D). In addition, DRD2 knockout in ARK2 cells did not completely abrogate the tumor-suppressor effect of ONC206, suggesting that other D2-like receptors independent pathways may play a role in modulating the effect of ONC206 in USC cells.

To further determine if the tumor-suppressive effect of ONC206 is mostly due to DRD2 antagonism, we examined the effect of DRD2 knockout on metabolic reprogramming and OXPHOS in USC cells. The results, again, showed that DRD2-KO cells had lower ATP and lactate production levels than those in the Control-KO cells, even without treatment with ONC206 (Figure S3E,F). Moreover, the decreases in ATP and lactate production levels following treatment with ONC206 were smaller in DRD2-KO cells than in Control-KO cells, suggesting that the effects of ONC206 on the mitochondrial ETC, OXPHOS, and glycolysis are disrupted in the absence of DRD2 (Figure 4B,C). These results support our hypothesis that DRD2 mediates the tumor-suppressive effect of ONC206 by suppressing OXPHOS and glycolysis in USC cells.

### 2.6. DRD2 Overexpression Is Associated with USC Progression and Patient Survival

To determine the role of DRD2 in USC progression, we first determined the expression level of endogenous DRD2 in USC cells ARK1, ARK2 and HEC50. We found that DRD2 mRNA level is overexpressed in USC cell lines compared to normal endometrial cells (Figure S4A).

We then analyzed TCGA RNA-seq data generated from endometrial cancer to investigate the differential expression of DRD2 in different subtypes. The results showed that DRD2 RNA expression is significantly higher in USC than in EEC (Figure S4B). Next, immunohistochemical analysis of DRD2 was performed in endometrial tissue samples from our own patient cohort. The results confirmed that USC expressed significantly higher DRD2 protein levels than did EEC, particularly low-grade EEC (grades I and II), and normal endometrium (Figure 4D,E). Additionally, significantly higher DRD2 expression was also observed in short-term (<2 years) USC survivors compared with long-term (>5 years) USC survivors, suggesting that DRD2 has prognostic significance (Figure 4F).

### 2.7. ONC206 Synergizes with Paclitaxel for USC Cell Viability

**Figure 5:**
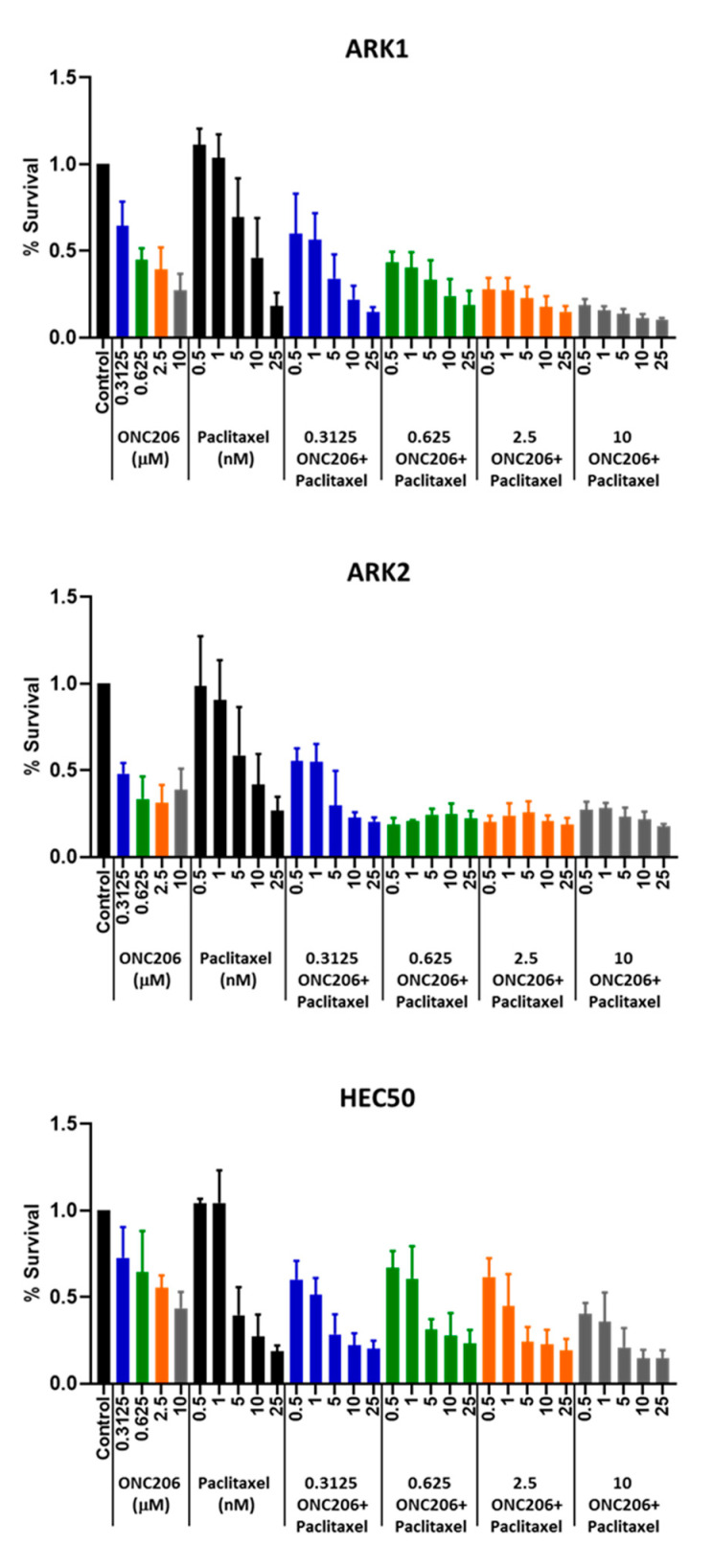
Effect of ONC206 in combination with paclitaxel on uterine serous cancer cell viability. ARK1, ARK2 and HEC50 cells were treated with ONC206 alone (0–10 μM) or in combination with paclitaxel (0–25 nM) for 72 h. ONC206 synergized with paclitaxel in reducing cell viability of USC cells. Three independent experiments were performed (mean ± standard deviation). The combination index was calculated using CompuSyn (combination index <1 indicates a synergistic effect).

Paclitaxel is one of the standard first-line treatment regimens for advanced USC in the clinic, and ONC201 was previously reported to synergize with paclitaxel in endometrial cancer [21]. Therefore, we hypothesized that ONC206 may have a similarly synergistic effect with paclitaxel on USC cell viability. USC cells ARK1, ARK2, and HEC50 were treated with ONC206 alone (0–10 μM) or in combination with paclitaxel (0–25 nM) for 72 h and cell viability was measured using the MTT assay. The results showed that ONC206 synergized with paclitaxel in reducing cell viability of USC cells, with combination index values <1. This suggests that the combination of ONC206 and paclitaxel can be a potential new treatment regimen for advanced USC (Figure 5 and Table 1).

## 3. Discussion

In the current study, we tested the novel orally bioavailable imipridone ONC206, a derivative of the first-in-class imipridone ONC201, for its anti-tumor effects and explored the underlying mechanism by which ONC206 suppresses the malignant phenotype of USC. Our findings demonstrated that ONC206 has a markedly higher potency compared to its parental compound ONC201 and exhibits significant induction of apoptosis in vitro. In an orthotopic mouse model for advanced USC, ONC206 treatment significantly reduced the tumor burden compared to either the control or ONC201 treatment, indicating ONC206 is more potent in suppressing the malignant phenotypes of USC. We demonstrated that inhibiting p38MAPK/ERK signaling pathway, and suppressing both glycolysis and oxidative phosphorylation (OXPHOS) in USC through antagonizing DRD2 is one of the potential mechanisms for the anti-tumor effect of ONC206.

As the founding member of imipridones, ONC201 is currently being tested in multiple clinical trials of hematological malignancies and solid tumors [15,22]. It is previously reported that ONC201 exerts the anti-tumor effects through the induction of TRAIL expression [23]. Therefore, ONC201 can promote apoptosis via the extrinsic cell death pathway. Our data revealed a similar but stronger effect of ONC206 on apoptosis induction compared to ONC201. However, we did not observe a significant induction of TRAIL in our tested USC cell lines in response to ONC206 treatment, indicating a distinct apoptotic pathway may be involved. Previous studies have confirmed that ONC201 is a direct competitive antagonist of dopamine receptors DRD2 and DRD3 [15,22]. Dopamine receptors are G-protein coupled receptors (GPCRs) and categorized into two subtypes: D1- and D2-like receptors [24]. DRD2 and DRD3 belong to the D2-like receptors and are normally associated with neurological diseases. Accumulating evidence has shown that simultaneous inactivation of the DRD2/3 signaling pathway can have therapeutic effects in cancer patients such as pancreatic cancer, as suggested by the anti-tumor effects of ONC201 [25,26,27]. Similarly, ONC206 was shown to be an antagonist for DRD2 and DRD3 as previously described [19]. This is supported by our data showing the anti-tumor effect of ONC206 is partially abrogated in DRD2-KO cells. Knockout DRD3 together with DRD2 may be needed to completely abrogate the ONC206 effect.

Recent studies also have demonstrated that ONC201 can bind to mitochondrial caseinolytic protease P (CIpP) and consequently lead to a reduction in mitochondrial ETC subunits, and impaired OXPHOS [28,29]. Based on our RPPA and Western blot analyses, we observed a similar reduction following ONC206 treatment. Besides, our results demonstrated that PGC-1α expression levels were significantly lower in ONC206-treated cells. PGC-1α is a transcription factor coactivator that influences a majority of cellular metabolic pathways [30]. The decrease in PGC-1α expression is likely due to inactivation of p38MAPK/ERK signaling pathway, which has been shown to increase the transcription of PGC-1α [31].

ONC206 inactivates p38MAPK and subsequently destabilizes and down-regulates PGC-1α and TFAM. PGC-1α has been shown to enhance OXPHOS and ROS degradation [32], and TFAM is the most well-known nuclear regulator in the mitochondrial transcription and replication machinery which belongs to the high mobility group (HMG) superfamily [33,34]. It is known that TFAM maintains the integrity of mitochondrial DNA (mtDNA) and therefore the loss of TFAM could cause mitochondrial damage and malfunction [35]. Downregulation of PGC-1α and TFAM by ONC206 might result in the disruption of mitochondrial ETC and thus inhibit OXPHOS, reduce ATP production and induce ROS levels as we observed in ONC206-treated cells (Figure S5).

## 4. Materials and Methods

### 4.1. Reagents

Small molecules ONC201 and ONC206 were provided by Oncoceutics Inc. (Philadelphia, PA, USA). Dimethyl sulfoxide (Fisher Scientific, Hampton, MH, USA) and sterile deionized waster were used as solvents to dissolve the molecules for in vitro and in vivo experiments, respectively.

### 4.2. Cell Lines and Culture Conditions

Human USC cell lines ARK1 and ARK2 (a gift from Dr. Alessandro D. Santin at Yale Cancer Center) and HEC50 (American Type Culture Collection, Manassas, VA, USA) were maintained in RPMI 1640 medium supplemented with 10% fetal bovine serum, and penicillin-streptomycin (Thermo Fisher Scientific, Waltham, MA, USA). All cell lines tested negative for mycoplasma contamination according to the MycoAlert mycoplasma detection kit (Lonza Group Ltd., Basel, Switzerland) upon thawing from frozen and all cell lines were authenticated by short tandem repeat profiling in the Characterized Cell Line Core Facility at The University of Texas MD Anderson Cancer Center.

### 4.3. MTT Assay

Cells were seeded on 96-well plate at 2000 cells per well, followed by incubation with gradient doses (0–10 μM) of ONC201 or ONC206 for 72 h. Cells were then incubated with 0.5 mg/mL 3-(4,5-dimethylthiazol-2-yl)-2,5-diphenyltetrazolium bromide (MTT; Sigma-Aldrich Co. St. Louis, MO, USA) in phosphate-buffered saline (PBS) for 2 h at 37 °C. The formazan that formed was then solubilized by adding dimethyl sulfoxide. The absorbance was read at 570 nm using a FLUOstar Galaxy plate reader (BMG Labtech, Offenburg, Germany). Cell viability curve and half-maximal inhibitory concentration (IC50) values were calculated using GraphPad Prism 8 (GraphPad Software, San Diego, CA, USA). The synergistic effect of the combination of ONC206 and paclitaxel on cell viability was determined using the combination index, calculated using CompuSyn (ComboSyn, Inc., Paramus, NJ, USA). A combination index <1 indicates synergism, =1 indicates an additive effect, and >1 indicates antagonism [36].

### 4.4. xCELLigence Real-Time Cell Analysis System

A real-time cell proliferation assay was performed using the xCELLigence system (ACEA Biosciences, Inc., San Diego, CA, USA). Cells were seeded on E-plate 16 at 5000 cells per well for 24 h before treatment with ONC206 (0.5 μM and 10 μM). Cell proliferation was monitored and recorded for 100 h after treatment with ONC206. A unit-less parameter termed the cell index was derived and used to represent the number of cells based on the measured relative change in electrical impedance that occurred in the presence and absence of cells in the wells.

### 4.5. Apoptosis Assay

Cells were treated with different concentrations of ONC201 and ONC206 for 72 h. Apoptosis was measured using the FITC Apoptosis Detection kit (BD Biosciences, San Jose, CA, USA) according to the manufacturer’s protocol. Cells were washed and stained with Annexin V-fluorescein isothiocyanate (FITC) and propidium iodide. Staining was then read using a Gallios flow cytometer (Beckman Coulter Inc., Brea, CA, USA).

### 4.6. Tumor Xenografts

Luciferase-labeled ARK1 cells (4 × 10^6^) were injected intraperitoneally into female athymic mice at the age of 6 weeks to establish tumors. After 2 weeks, the animals were randomized into three groups for treatment with PBS, ONC201, or ONC206; 100 mg/kg ONC201 or ONC206 was administered intraperitoneally twice per week for 6 weeks. The tumor volumes were measured and quantified using the IVIS-Lumina XR in vivo imaging system (Caliper Life Science, Inc., Hopkinton, MA, USA). Mice were euthanized using a carbon dioxide chamber followed by cervical dislocation after 6 weeks of treatment. Tumor tissues were fixed in formalin and processed for immunohistochemistry. The described animal procedures were reviewed and approved by the institutional animal care and use committee at MD Anderson.

### 4.7. Immunohistochemistry

Immunolocalization of DRD2 was performed using formalin-fixed, paraffin-embedded uterine tumor sections obtained from patients with endometrioid endometrial cancer (EEC) or USC. Slides containing the sections were stained with commercially available anti-DRD2 (1:200; 55084–1-AP; Proteintech Group Inc., Rosemont, IL, USA). Human uterine tumor tissue sections from patients with EEC and USC were obtained from the uterine cancer repository of the Department of Gynecologic Oncology and Reproductive Medicine under protocols approved by the institutional review board (IRB) at MD Anderson. The IRB code is LAB02-188. Informed consent was obtained from all patients. Immunolocalization of Ki-67 was performed using formalin-fixed, paraffin-embedded uterine tumor sections obtained from the uterine cancer-bearing athymic mice treated with ONC201, ONC206, or PBS. Slides containing the sections were stained with commercially available anti-Ki-67 (1:200; BDB550609; BD Biosciences). In brief, tissue sections were deparaffinized and dehydrated, and antigen retrieval was performed in sodium citrate buffer (pH 6.0) with a microwave at 95 °C for 15 min. After blocking, sections were incubated with the primary antibody at room temperature for 1 h, washed twice with PBS, and incubated with biotinylated mouse secondary antibody for 1 h. Target protein expression in the tumor sections was visualized using a Betazoid 3,3′-diaminobenzidine chromogen kit (Biocare Medical, Concord, CA, USA). Images of three fields of each slide were taken at ×20 magnification.

### 4.8. TUNEL Assay

In situ apoptosis was measured using the ApopTag Plus Peroxidase in Situ Apoptosis Kit (MilliporeSigma, Burlington, MA, USA) according to the manufacturer’s protocol. In brief, formalin-fixed, paraffin-embedded uterine tumor sections obtained from the uterine cancer-bearing athymic mice treated with ONC201, ONC206, or PBS were first deparaffinized and dehydrated, and antigen retrieval was performed in sodium citrate buffer (pH 6.0) with a microwave at 95 °C for 15 min. Proteinase K (20 μg/mL) was then applied to the sections at ambient temperature for 15 min, followed by hydrogen peroxide quench. Next, sections were incubated with equilibration buffer and then with working strength TdT enzyme at 37 °C for 1 h. After incubation with stop buffer for 10 min, the sections were incubated with anti-digoxigenin peroxidase conjugate at ambient temperature for 30 min. Sections were then developed with peroxidase substrate and counterstained with 0.5% methyl green before washing with N-butanol, and the sections were mounted with coverslips.

### 4.9. Reverse Phase Protein Array (RPPA)

Cell lysates were extracted in lysis buffer (1% Triton X-100, 50 mM HEPES pH 7.4, 150 mM NaCl, 1.5 mM MgCl_2_, 1 mM EGTA, 100 mM NaF, 10 mM NaP_2_O_7_, 1 mM Na_3_VO_4_ and 10% glycerol) containing freshly added protease and phosphatase inhibitors provided by the Functional Proteomics RPPA Core Facility at MD Anderson. Cell lysates were then denatured by 1% SDS (with β-mercaptoethanol) and diluted in five 2-fold serial dilutions in dilution lysis buffer. Serial diluted lysates were arrayed on nitrocellulose-coated slides (Grace Bio-Labs, Bend, OR, USA) by an Aushon 2470 Arrayer (Aushon BioSystems Inc., Billerica, MA, USA). A total of 5808 array spots were arranged on each slide including the spots corresponding to serial diluted “Standard Lysates,” and positive and negative controls were prepared from mixed cell lysates or dilution buffer, respectively. Each slide was probed with a validated primary antibody plus a biotin-conjugated secondary antibody. The signal obtained was amplified using a Dako Cytomation-Catalyzed System (Dako, Santa Clara, CA, USA) and visualized by DAB colorimetric reaction. The slides were scanned, analyzed, and quantified using customized software to generate spot intensity. Each dilution curve was fitted with a logistic model—“Supercurve Fitting,” developed by the Department of Bioinformatics and Computational Biology at MD Anderson (http://bioinformatics.mdanderson.org/OOMPA).

### 4.10. Western Blot Analysis

Cell extracts were prepared in RIPA buffer (Boston Bioproducts, Inc., Ashland, MA, USA) containing complete protease inhibitor cocktail (Thermo Fisher Scientific). Proteins were separated on SDS-polyacrylamide gels and electrophoretically transferred to an Immobilon polyvinylidene fluoride membrane (EMD Millipore, Billerica, MA, USA). The membranes were incubated with primary antibodies overnight at 4 °C and then incubated with appropriate horseradish peroxidase-conjugated secondary antibodies at 1:10,000 dilution (Thermo Fisher Scientific) at ambient temperature for 1 h. Signals were developed using ECL chemiluminescence detection reagents (Denville Scientific Inc., Holliston, MA, USA) and visualized on X-ray film (Denville Scientific Inc.). The primary antibodies used are listed in Table S3.

### 4.11. ATP Production Assay

ATP production was measured using the ATP determination kit (Thermo Fisher Scientific) according to the manufacturer’s protocol. In brief, after 48 h of treatment with ONC206, cells were lysed with 1% NP-40. Supernatant of the cell lysate was mixed with an equal volume of the reaction solution (0.1 M DTT, 10 mM D-luciferin, 5 mg/mL firefly luciferase and 20× reaction buffer) for 15 min at ambient temperature before luminescence was measured using a FLUOstar Galaxy plate reader (BMG Labtech). The amount of ATP produced was determined using a standard curve method with known ATP concentrations.

### 4.12. Cytochrome c Oxidase Activity Assay

Cytochrome c oxidase activity in intact mitochondria isolated from cells treated with ONC206 was measured using the cytochrome c oxidase activity assay kit (Sigma-Aldrich Co.) according to the manufacturer’s protocol. In brief, ferrocytochrome c substrate solution was added to the isolated mitochondria and the initial absorbance at 550 nm was measured immediately. The decrease in absorbance at 550 nm, which was caused by the oxidation of ferrocytochrome c to ferricytochrome c by cytochrome c oxidase, was followed for a minute using a kinetic program of the DS-11 FX+ spectrophotometer (DeNovix, Wilmington, DE, USA).

### 4.13. Lactate Production Assay

Intracellular lactate production was measured using the Lactate-Glo™ assay kit (Promega Corp., Madison, WI, USA) according to the manufacturer’s protocol. In brief, cells were plated on a 96-well plate overnight before 24 h of treatment with ONC206. Cells were then washed and incubated with the inactivation (0.6 N hydrochloric acid) and neutralization (1 M Tris base) solutions to inactivate endogenous enzyme activity. An equal volume of lactate detection reagent (luciferin detection solution, reductase, reductase substrate, lactate dehydrogenase and NAD) was added to the cells and incubated at ambient temperature for 1 h before luminescence was measured using a FLUOstar Galaxy plate reader (BMG Labtech). The luminescent signal was proportional to the amount of lactate in the sample.

### 4.14. Reactive Oxygen Species (ROS) Detection Assay

The intracellular ROS level was measured using the ROS detection assay kit (BioVision Inc., Milpitas, CA, USA) according to the manufacturer’s protocol. In brief, cells were plated on a 96-well plate overnight before treatment with ONC206. Cells were then washed and incubated with 2′,7′-dichlorodihydrofluorescein diacetate at 37 °C in the dark for 45 min. Upon action of cellular esterases and oxidation by ROS, a highly fluorescent product was formed. It was detected using a FLUOstar Galaxy plate reader (BMG Labtech) at an excitation wavelength of 485 and an emission wavelength of 520 nm. The fluorescence intensity was proportional to the ROS level in the sample.

### 4.15. DRD2 Knockout Using CRISPR-Cas9

ARK2 cells were co-transfected with DRD2 CRISPR/Cas9 KO plasmid (sc-400235-KO-2, Santa Cruz Biotechnology, Inc., Dallas, TX, USA) and DRD2 HDR plasmid (sc-400235-HDR-2, Santa Cruz Biotechnology, Inc.), and selected for with puromycin to generate DRD2 knockout (DRD2-KO) cells. Control CRISPR/Cas9 plasmid (sc-418922, Santa Cruz Biotechnology, Inc.) was also transfected to generate control knockout (Control-KO) cells as a negative control. Single DRD2-KO and Control-KO cells, which were RFP positive, were isolated using a cell sorting technique. The knockout efficiency was verified using quantitative reverse transcription PCR analysis.

### 4.16. Quantitative Reverse Transcription PCR Analysis

Total RNA was extracted from cultured cells using TRI reagent (Molecular Research Center, Cincinnati, OH, USA) and 0.5 μg of total RNA was used to synthesize the first strand of cDNA using the ImProm-II Reverse Transcription System (Promega Corp.). Pre-designed, FAM-labeled human DRD2 (Hs00241436_m1) and VIC-labeled human GAPDH TaqMan gene expression assays (Life Technologies Corp.) were used in the real-time PCR analysis. The relative standard curve method (2^−ΔΔCt^) was used to determine the relative mRNA expression, using GAPDH as the reference.

### 4.17. Analysis of DRD2 Expression in The Caner Genome Atlas (TCGA) Data

mRNA sequencing data for the UCEC dataset from TCGA (data version 2016-01-28) was downloaded from the Broad Institute TCGA Genome Data Analysis Center (http://firebrowse.org/). The difference in DRD2 expression between EEC (*n* = 409) and pure USC (*n* = 114) was determined using the Mann–Whitney U test.

### 4.18. Statistical Analysis

SPSS software, version 24 (IBM Corp. Armonk, NY, USA), and GraphPad Prism 6 (GraphPad software) were used to perform statistical tests. Data are presented as mean ± standard deviation unless otherwise specified. A two-tailed Student t test was used to test differences in sample means for data with normally distributed means. The Mann–Whitney U test was used for non-parametric data as appropriate. A *p*-value of <0.05 was considered statistically significant.

## 5. Conclusions

In summary, our study reveals that ONC206 exhibits strong therapeutic efficacy in USC in vitro and in mouse models, and demonstrates the molecular mechanism by which ONC206 suppresses the malignant phenotype of USC. Knockout experiments demonstrated a DRD2-dependent anti-tumor effect of ONC206. Immunolocalization of DRD2 showed the prognostic significance of DRD2 particularly in USC. These findings suggest that ONC206 may have higher efficacy in the treatment of USC, which express high levels of DRD2, and DRD2 may be used a predictive marker for response to ONC206 treatment. Further investigation of the efficacy of ONC206 in patients with USC is warranted.

## Figures and Tables

**Table 1:** Combination Index of ONC206 in combination with paclitaxel in uterine serous cancer cells.

ONC206 (μM)	Paclitaxel (nM)	Combination Index		
		ARK1	ARK2	HEC50
0.3125	0.5	1.180	0.179	0.613
	1	0.889	0.267	0.527
	5	0.431	0.658	0.496
	10	0.488	0.480	0.582
0.625	0.5	0.464	0.224	1.702
	1	0.389	0.379	1.292
	5	0.484	0.164	0.619
	10	0.554	0.204	0.919
2.5	0.5	0.321	0.441	2.637
	1	0.335	0.301	0.592
	5	0.392	0.432	0.371
	10	0.463	0.177	0.627
10	0.5	0.347	0.111	0.801
	1	0.225	0.184	0.559
	5	0.281	0.117	0.304
	10	0.345	0.398	0.284

## Supplementary Materials

The following are available online at https://www.mdpi.com/2072-6694/12/9/2436/s1. Figure S1: Effect of ONC201 and ONC206 on body weight of C57BL/6 mice, Figure S2: Effect of ONC206 on pro-apoptotic protein expressions, Figure S3: Role of DRD2 in mediating the suppressive effect of ONC206 on USC cells, Figure S4: DRD2 RNA expression in different endometrial cell lines and tissues, Figure S5: Schematic summarizing the proposed pathways in which ONC206 is involved in suppressing the malignant phenotype of uterine serous cancer, Table S1: Half-maximal inhibitory concentration (IC50) values for uterine serous cancer cells after treatment with ONC201 or ONC206 for 72 h, Table S2: Fold change of protein expression in ONC206-treated ARK1 cells, as measured using reverse phase protein array. Table S3: Primary antibodies used for Western blot analyses.
