# Correction: Hu, W., et al. Targeting Dopamine Receptor D2 by Imipridone Suppresses Uterine Serous Cancer Malignant Phenotype. *Cancers* 2020, *12*, 2436

**DOI:** 10.3390/cancers13040623

**Published:** 2021-02-04

**Authors:** Wen Hu, Li Zhang, Sammy Ferri-Borgogno, Suet-Ying Kwan, Kelsey E. Lewis, Han T. Cun, Tsz-Lun Yeung, Pamela T. Soliman, Rohinton S. Tarapore, Joshua E. Allen, Xinyuan Guan, Karen H. Lu, Samuel C. Mok, Chi-Lam Au-Yeung

**Affiliations:** 1 Department of Gynecologic Oncology and Reproductive Medicine, The University of Texas MD Anderson Cancer Center, Houston, TX 77030, USA; whu2@mdanderson.org (W.H.); lzhang25@mdanderson.org (L.Z.); sferri@mdanderson.org (S.F.-B.); hcun@mdanderson.org (H.T.C.); tyeung@mdanderson.org (T.-L.Y.); psoliman@mdanderson.org (P.T.S.); khlu@mdanderson.org (K.H.L.); scmok@mdanderson.org (S.C.M.); 2 State Key Laboratory of Oncology in South China and Collaborative Center for Cancer Medicine, Sun Yat-Sen University Cancer Center, Guangzhou 510060, China; xyguan@hkucc.hku.hk; 3 Department of Molecular and Cellular Biology, The University of Texas MD Anderson Cancer Center, Houston, TX 77030, USA; skwan1@mdanderson.org; 4 Department of Obstetrics and Gynecology, The University of Texas Medical Branch at Galveston, Galveston, TX 77555, USA; kelewis@utmb.edu; 5 Oncoceutics Inc., Philadelphia, PA 19104, USA; rohinton.tarapore@oncoceutics.com (R.S.T.); josh.allen@oncoceutics.com (J.E.A.)

In the original article, there was a mistake in Figure 2B as published [1]. Two images from the same mouse with different luminescence signals were presented.

When we image mice, we usually do it twice at different exposure time to make sure we capture any luminescence signals. All the mouse images presented in the manuscript, and the values of luminescence signals used for quantification, are from the same exposure time. However, the two mouse images highlighted in the PBS group in Figure 2B were indeed from the same mouse that were captured at different exposure times. That is why the luminescence signals of the two are different. That is a manual error when processing a large number of images. The authors have double-checked all of them and replaced the panel with a correct image. Another mistake is also fixed with one other image being swapped between the PBS and ONC201 groups. Nevertheless, these did not affect the quantification in Figure 2C, as the calculation was from values generated from the imaging software, which is independent to the process of putting the images together manually.

The corrected Figure 2B appears below: 


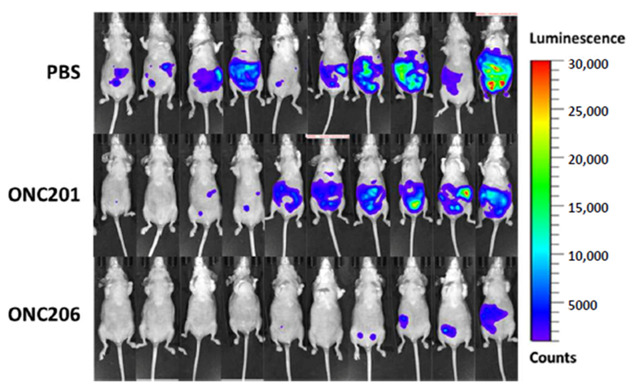


The authors apologize for any inconvenience caused and state that the scientific conclusions are unaffected. All the authors have checked and agreed with the corrected paper content. The original article has been updated.
