# Supplementary material for: Targeting Dopamine Receptor D2 by Imipridone Suppresses Uterine Serous Cancer Malignant Phenotype
Source: Cancers (Basel). 2020 Aug 27;12(9):2436. doi: 10.3390/cancers12092436 (PMC7563948; doi:10.3390/cancers12092436)
Supplement: Supplementary file 1 [file cancers-12-02436-s001.zip › cancers-869856-supplementary/cancers-869856-supplementary-author proofed.pdf]

Supplementary Figure 1.

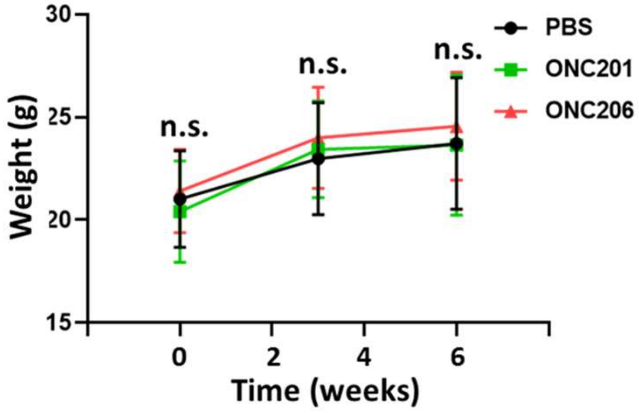

Figure S1. Effect of ONC201 and ONC206 on body weight of C57BL/6 mice. No significant difference in body weight was observed in mice treated with 100 mg/kg ONC201 or ONC206 twice per week for 6 weeks compared with the control phosphate-buffered saline (PBS) group. n. s.=not significant ($p>0.05$).

Supplementary Figure 2.

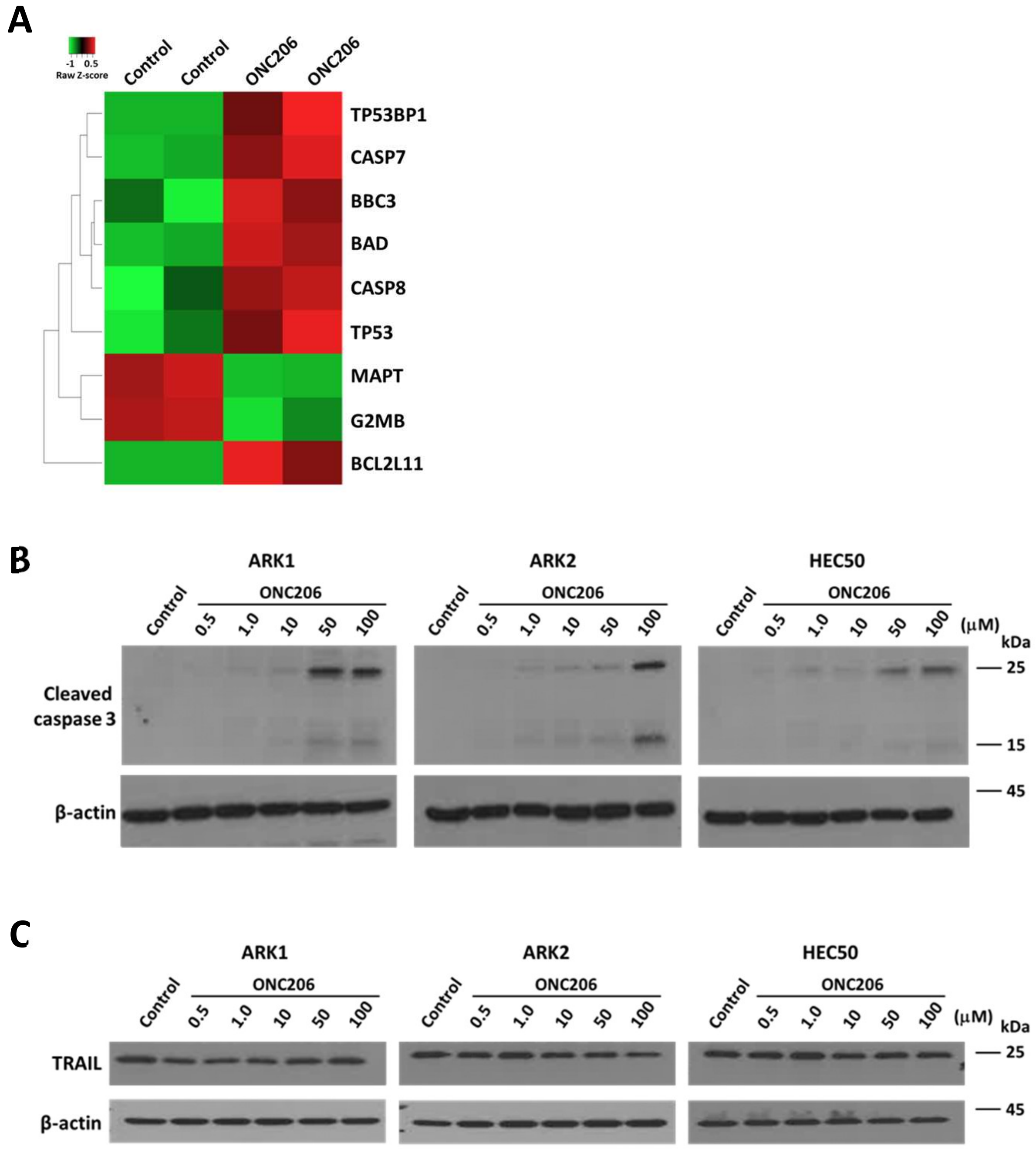

Figure S2. Effect of ONC206 on pro-apoptotic protein expression. (**A**) Heat map obtained using reverse phase protein array analysis shows differentially expressed proteins related to apoptosis in ARK1 cells with (n=2) or without (n=2) 48 hours of treatment with 50μM ONC206. (**B**) and (**C**) Western blot analyses show (**B**) increased cleaved caspase 3 protein level and (**C**) no significant change in TRAIL protein level in ONC206-treated ARK1, ARK2, and HEC50 cells compared with control cells without treatment. β-actin served as a loading control. Three independent experiments were performed.

Supplementary Figure 3.

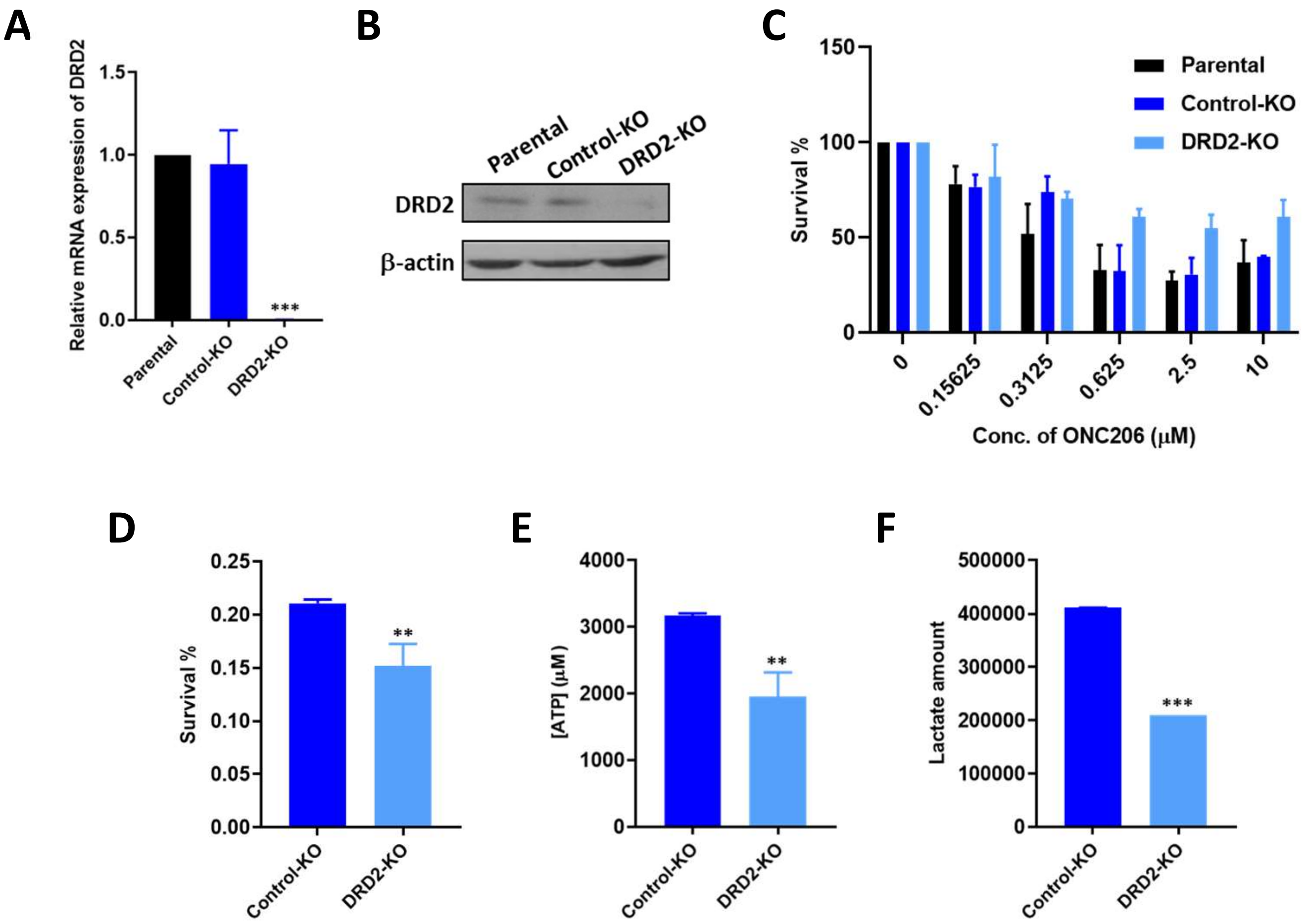

Figure S3. Role of DRD2 in mediating the suppressive effect of ONC206 in USC cells. (**A**) Quantitative reverse transcription PCR and (**B**) Western blot analyses showed that DRD2 was successfully knocked out in ARK2 cells. β-actin served as a loading control. Parental; Parental ARK2 cells. (**C**) No significant difference in cell viability was observed between parental ARK2 cells and Control-knockout (KO) ARK2 cells, whereas DRD2-KO cells were more resistant to ONC206 than Control-KO or parental ARK2 cells (Parental). Three independent experiments were performed (mean ± standard deviation). (**D-F**) Decrease in (**D**) cell viability, (**E**) ATP production, and (**F**) lactate secretion in DRD2-KO cells compared with Control-KO cells. Results were averaged from three independent experiments and are shown as mean ± standard deviation. ***p<0.001, **p<0.01, two-tailed Student t test.

Supplementary Figure 4.

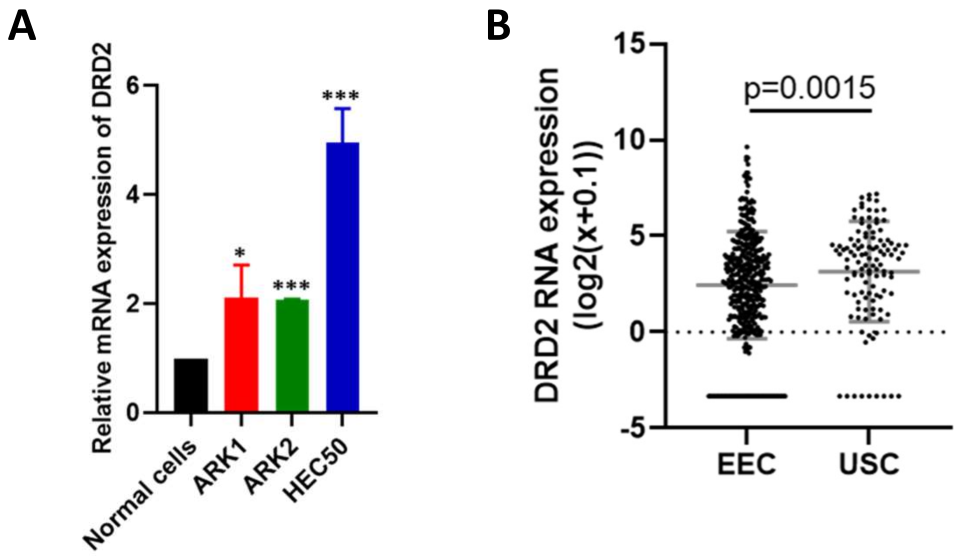

Figure S4. DRD2 RNA expression in different endometrial cell lines and tissues. (**A**) DRD2 mRNA level is overexpressed in uterine serous cancer (USC) cell lines compared to normal endometrial cells as measured by quantitative reverse transcription PCR analysis. Results were averaged from three independent experiments and are shown as mean ± standard deviation. ***p<0.001, *p<0.05, two-tailed Student t test. (**B**) TCGA RNA-seq data shows that DRD2 RNA expression is significantly higher in USC than in endometrioid endometrial cancer (EEC). EEC, n=409; USC, n=114. p=0.0015, Mann-Whitney U test.

Supplementary Figure 5.

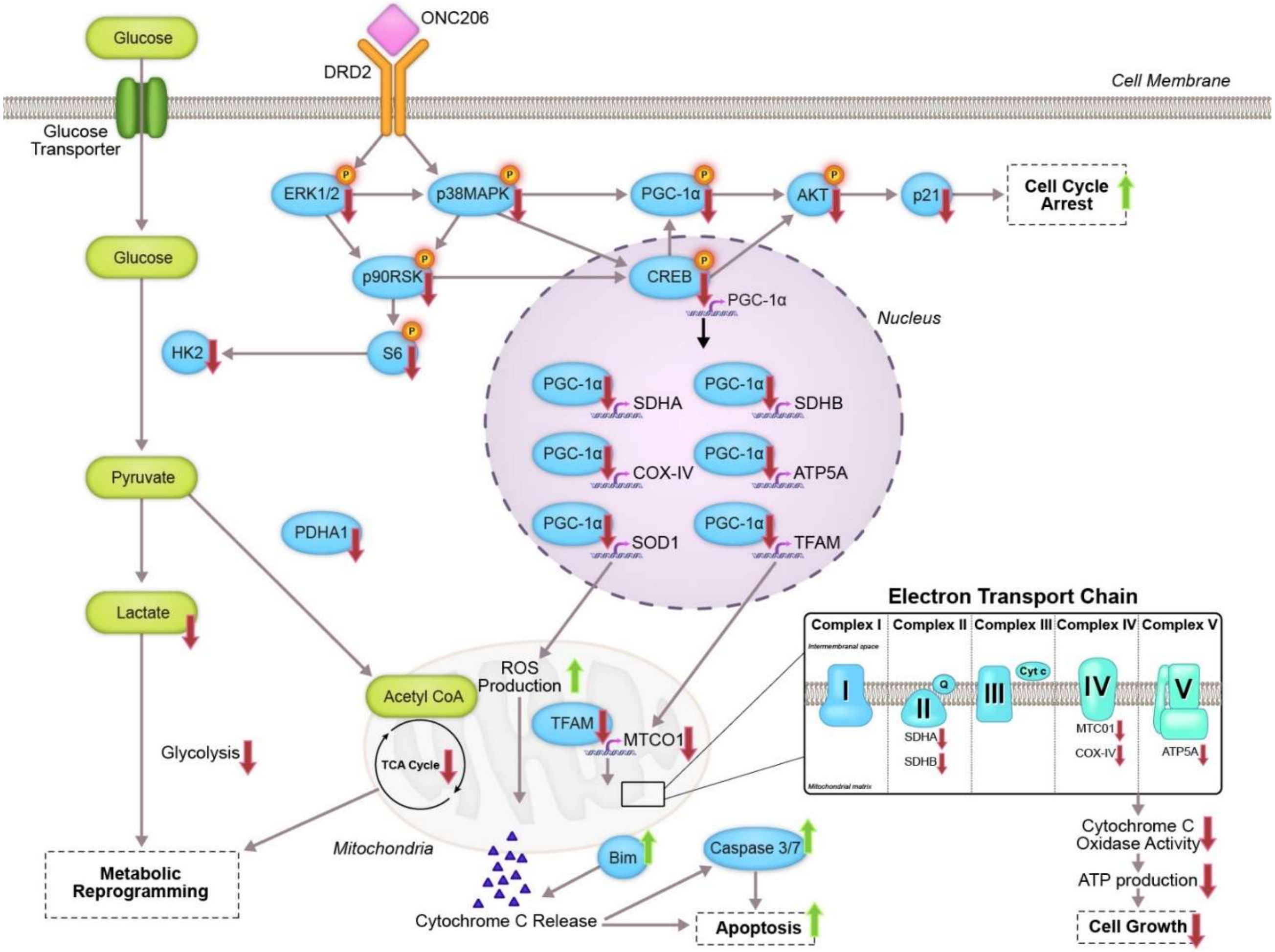

Figure S5. Schematic summarizing the proposed pathways in which ONC206 is involved in suppressing the malignant phenotype of uterine serous cancer. ONC206 binds with DRD2 to inactivate the downstream p38MAPK/ERK signaling network that leads to metabolic reprogramming and suppression of mitochondrial protein functions. These subsequently induce apoptosis, and reduce cell growth in uterine serous cancer cells. ROS, reactive oxygen species.

Table S1. Half-maximal inhibitory concentration (IC50) values for uterine serous cancer cells after treatment with ONC201 or ONC206 for 72 hours

Drug	IC50 (mM)		
	ARK1	ARK2	HEC50
ONC201	2.9±0.9	1.7±0.3	1.3±0.5
ONC206	0.3±0.03	0.16±0.003	0.34±0.2

Table S2. Fold change of protein expression in ONC206-treated ARK1 cells, as measured using reverse phase protein array

Gene name	Antibody name	Fold change (ONC206 treated/untreated)
MT-CO1	MTCO1	0.26228096
TFAM	TFAM	0.355846774
MAPK1/MAPK3	MAPK_pT202_Y204	0.417541537
RPS6KA1	p90RSK_pT573	0.55932949
SDHB	Complex-II-Subunit	0.588202023
SDHA	SDHA	0.593109831
TUFM	TUFM	0.603037065
RPS6	S6_pS235_S236	0.647703155
RPS6	S6_pS240_S244	0.661827971
ATP5F1A	ATP5A	0.666325428
AKT2	Akt2_pS474	0.667513954
ESRRA	ERRalpha	0.697072142
CDKN1A	p21	0.702952718
HK2	Hexokinase-II	0.739747015
COX4I1	Cox-IV	0.758993242
SOD1	SOD1	0.808422411
AKT1/2/3	Akt_pS473	0.820281891
PDHA1	PDHA1	0.843392938
AKT1/2/3	Akt	0.849420154
AKT2	Akt2	0.860281514
AKT1	Akt1_pS473	0.910276122
TP53	p53	1.26896059
BCL2L11	Bim	1.56169375
CASP7	Caspase-7-cleaved	1.591854094

Table S3. Primary antibodies used for Western blot analyses

Antibody	Dilution	Company	Catalog no.
p-ERK1/2 (T202/Y204)	1:1000	Cell Signaling Technology	9101
ERK1/2	1:1000	Cell Signaling Technology	9102
p-p38MAPK (T180/Y182)	1:1000	Cell Signaling Technology	4511
p38MAPK	1:1000	Cell Signaling Technology	8690
p-S6 (S240/S244)	1:1000	Cell Signaling Technology	5367
S6	1:1000	Cell Signaling Technology	2217
PGC-1α	1:1000	Novus Biologicals	NBP1-04676
TFAM	1:1000	Cell Signaling Technology	7495
SDHA	1:1000	Cell Signaling Technology	11998
MT-CO1	1:1000	Thermo Fisher Scientific	459600
COX-IV	1:1000	Cell Signaling Technology	4850
SOD1	1:1000	Cell Signaling Technology	4266
HK2	1:1000	Cell Signaling Technology	2867
PDHA1	1:1000	Cell Signaling Technology	3205
p21	1:1000	Santa Cruz Biotechnology Inc.	sc-397
TRAIL	1:1000	Cell Signaling Technology	3219
Cleaved caspase 3	1:1000	Cell Signaling Technology	9661
β-actin	1:5000	Sigma-Aldrich Co.	clone AC-15
