# Supplementary figures and images for: Targeting Dopamine Receptor D2 by Imipridone Suppresses Uterine Serous Cancer Malignant Phenotype
Source: Cancers (Basel). 2020 Aug 27;12(9):2436. doi: 10.3390/cancers12092436 (PMC7563948; doi:10.3390/cancers12092436)

Figure 3b.

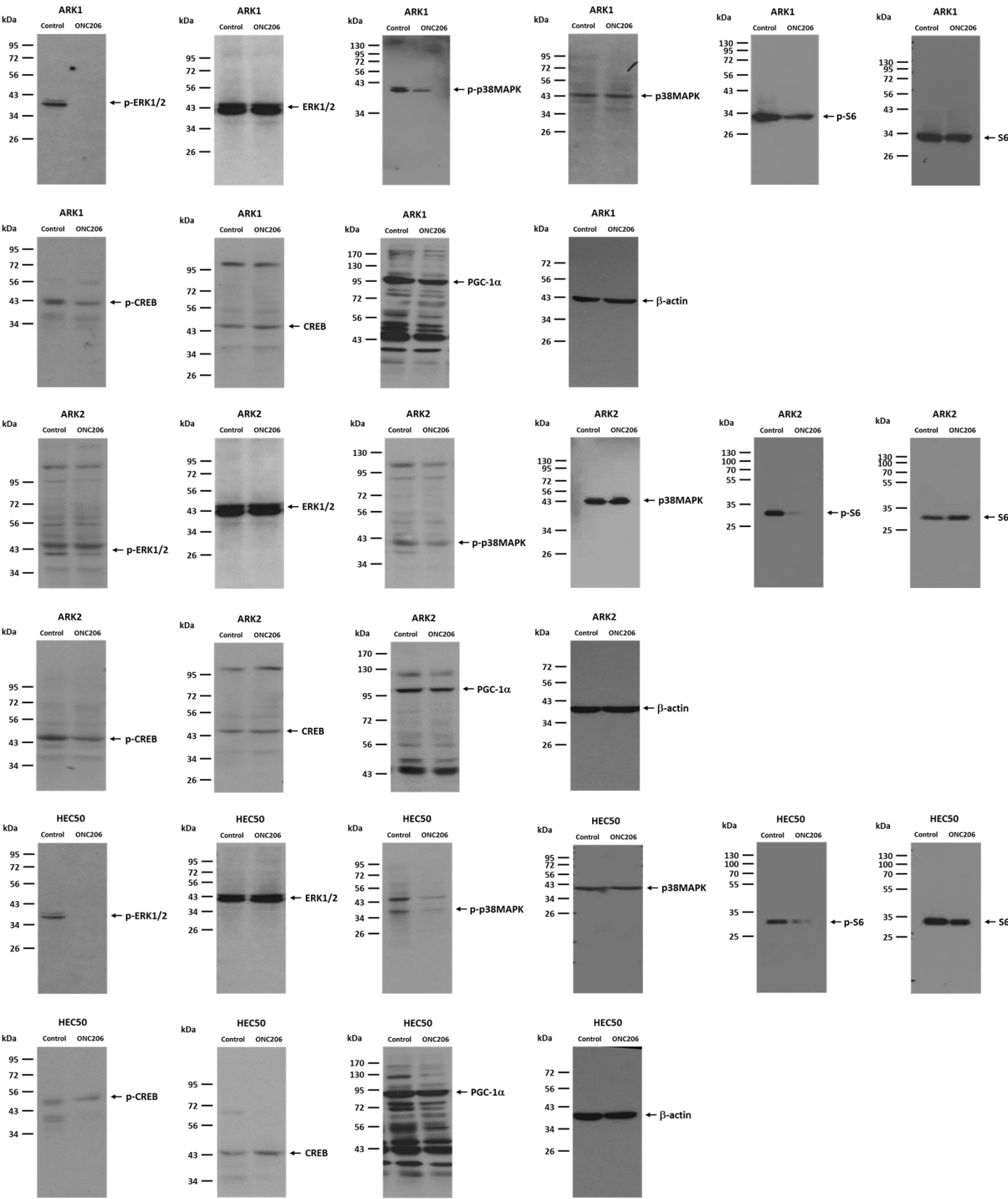

Figure 3c.

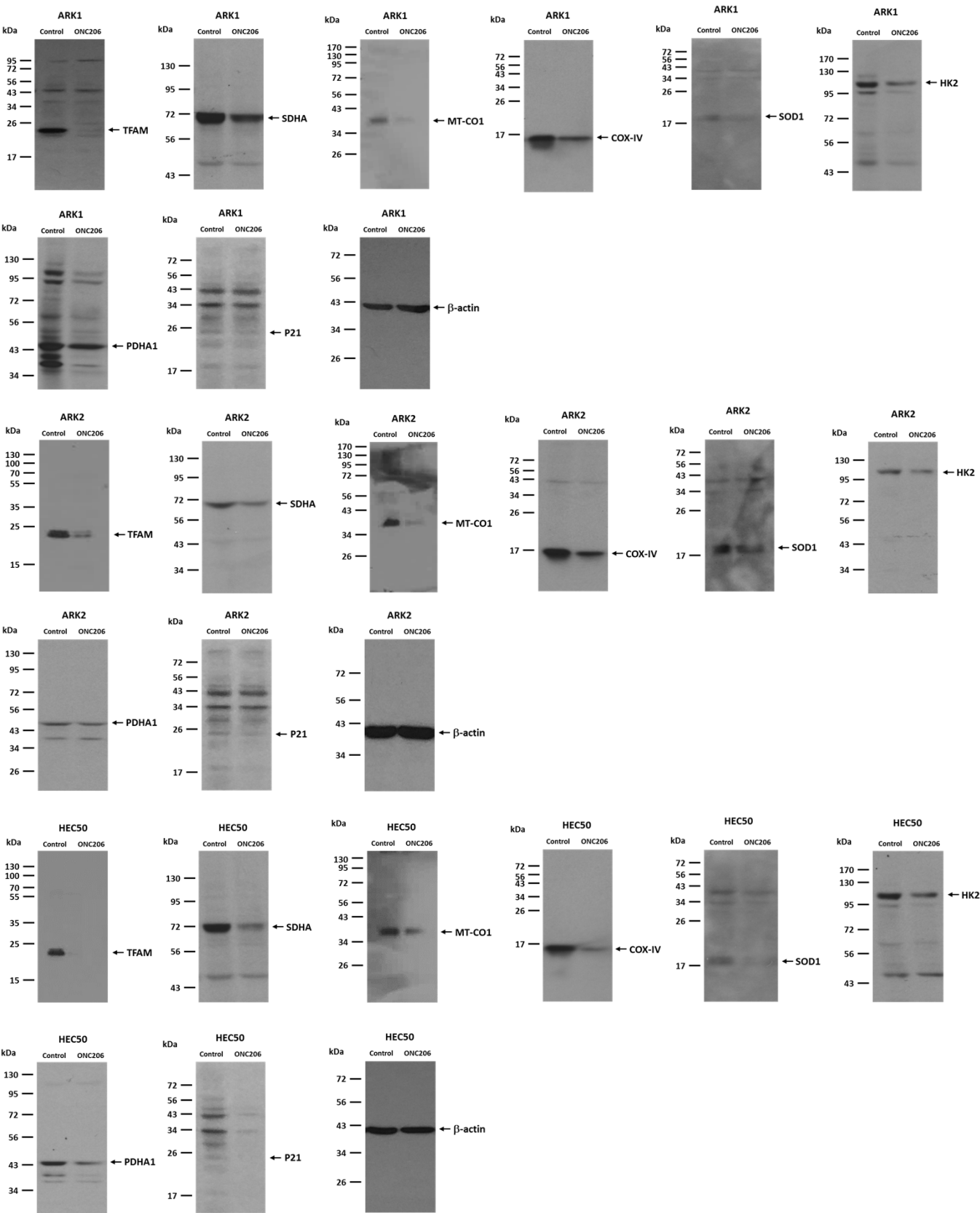

Figure S2b.

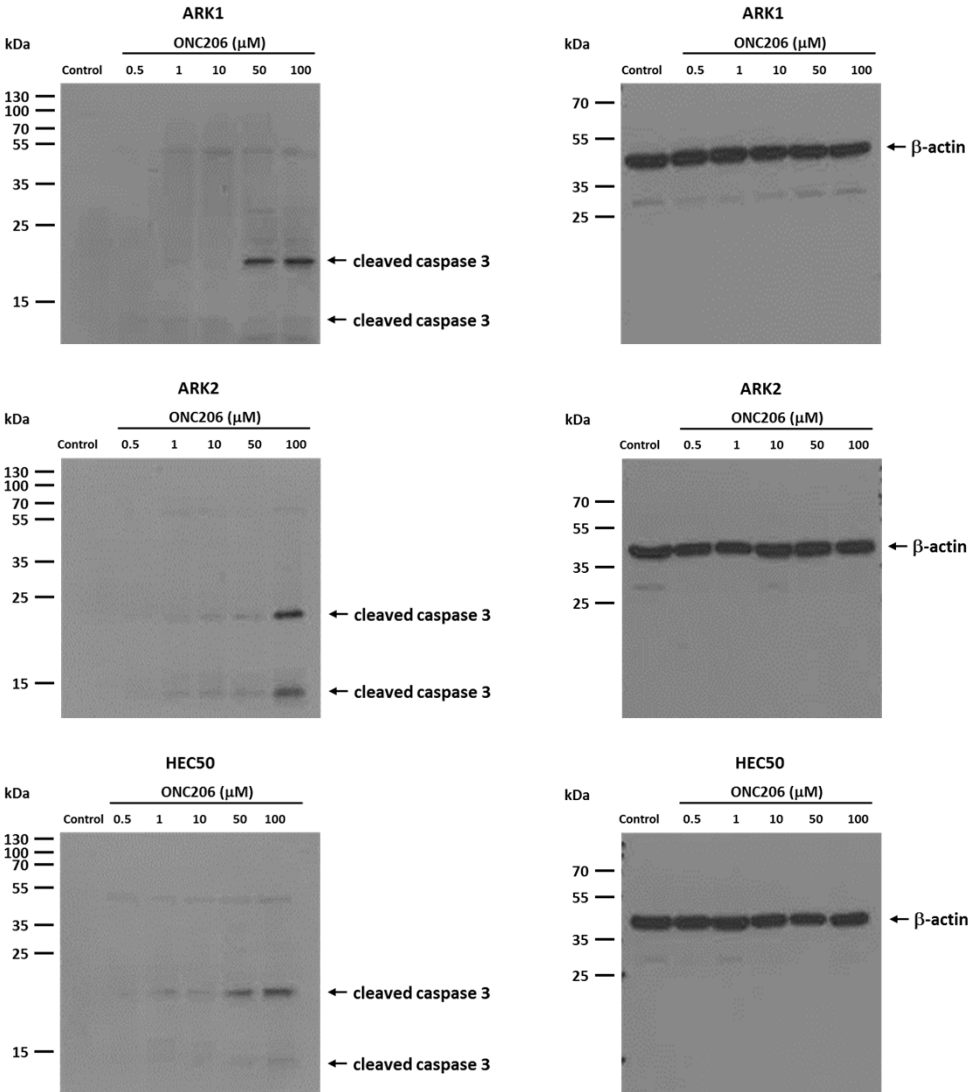

Figure S2c.

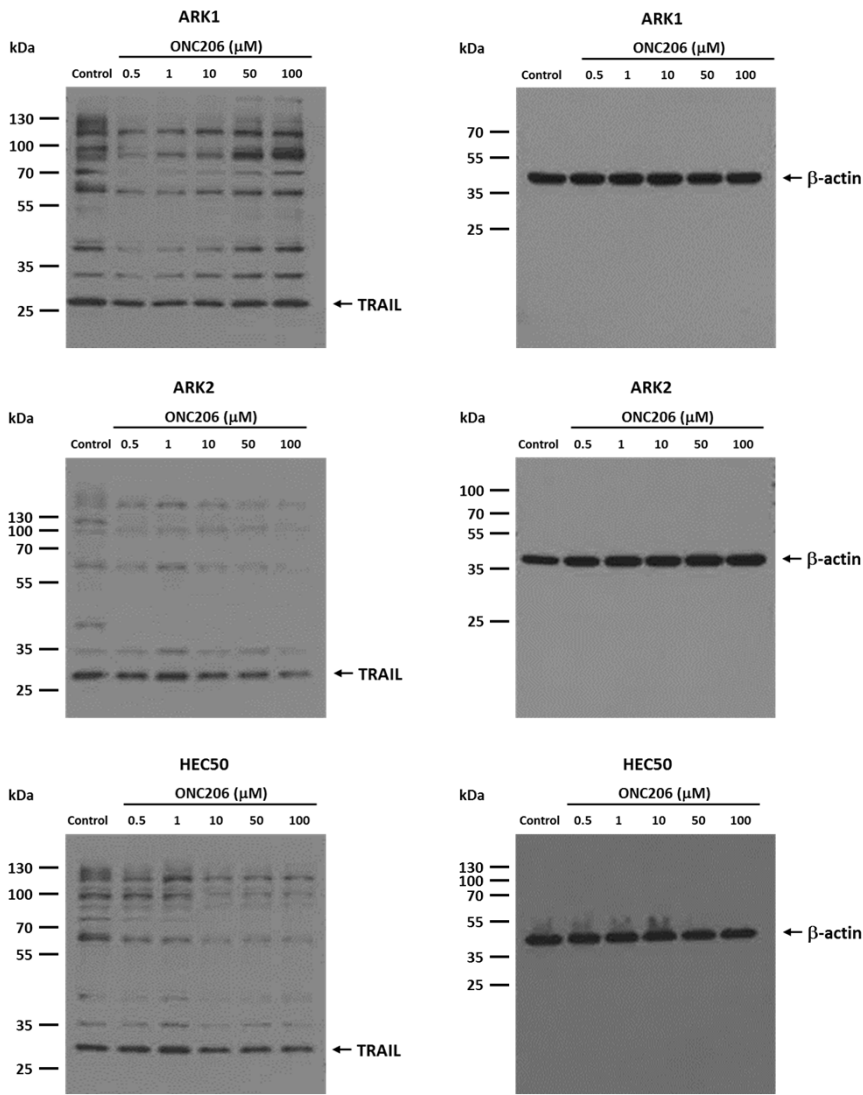

Supplement: Supplementary file 1 [file cancers-12-02436-s001.zip › cancers-869856-supplementary/cancers-869856-original_images.pdf]
